# Natural daylight restricted to twilights delays the timing of testicular regression but does not affect the timing of the daily activity rhythm of the house sparrow (*Passer domesticus*)

**DOI:** 10.1186/1740-3391-4-5

**Published:** 2006-03-23

**Authors:** Amit K Trivedi, Sangeeta Rani, Vinod Kumar

**Affiliations:** 1 Department of Zoology, University of Lucknow, Lucknow 226 007, India

## Abstract

**Background:**

A stable and systematic daily change in light levels at dawn and dusk provides the most reliable indicator of the phase of the day. It is likely that organisms have evolved mechanisms to use these twilight transitions as the primary zeitgeber to adjust their circadian phases. In this study, we investigated under natural illumination conditions the effects of daylight exposure restricted to twilights on the timing of testicular regression and locomotor activity of the house sparrow (*Passer domesticus*), which possesses a strongly self-sustaining circadian system.

**Methods and results:**

Two experiments were performed on adult male house sparrows. Beginning in the third week of April, the first experiment examined whether exposure to natural daylight only during twilights influenced the timing of testicular regression and concomitant changes in testosterone-dependent beak color of reproductively mature sparrows. Interestingly, there was a significant delay in testicular regression and depigmentation of the beak in sparrows exposed to natural daylight (NDL) only during twilights as compared to those exposed to NDL all day. The second experiment examined twice in the year, around the equinoxes (March and September), the effects of exposure to twilights only on the daily activity rhythm of sparrows kept in an outdoor aviary. Five of 7 birds continued exhibiting entrained activity rhythms when exposed only to twilights (NDL minus day light from sunrise to sunset) in September, but not in March. Both in NDL and twilight conditions, March birds had significantly lower activity counts than September birds.

**Conclusion:**

Exposure to natural daylight only during twilights delayed the timing of testicular regression and concomitant depigmentation of the beak but did not affect the daily activity rhythm in male sparrows. This suggests that daily twilights can serve as cues for regulation of the circadian activity rhythm but not for the photoperiodic regulation of testicular cycle in the house sparrow.

## Introduction

The adaptive value of biological rhythms is tied at least in part to their being synchronized to the right phases of the external cycle, which in most cases is the light-dark (LD) cycle of the environment. In fact, a stable and systematic daily change in light characteristics at dawn or dusk times (twilight times) serves as the most reliable indicator of the phase of the day [1]. Therefore, it is likely that organisms have evolved mechanisms to use twilight transition times as the primary zeitgeber to adjust their circadian phases [2]. Despite inter- and intra-species differences in the length of endogenous circadian periods (τ), in nature the discrepancy between τ and T is relatively small and is corrected for in the twilight zones, either at dawn (if τ >T) or at dusk (if τ < T), by advancing or delaying the activity, respectively [2].

The daily pattern of the activity-rest cycle has been described in numerous captive and free-living vertebrates exposed to cycles of natural illumination at different latitudes during different seasons [3,4]. However, these studies have not specifically addressed the effects of exposure to daylight restricted to twilights on the daily activity rhythms. The effect of twilights alone on the regulation of photoperiod-induced testicular cycles has not been investigated. Studies on the effects of twilights on the circadian system have used only simulated twilight transitions in laboratory conditions [5-13].

Extensive studies on the house sparrow (*Passer domesticus*) inhabiting temperate latitudes (40° N and higher) have established that (i) the sparrow possesses a strongly self-sustaining circadian system as revealed by the characteristics of its behavioral and pineal melatonin rhythms [14,15], and (ii) photoperiodic regulation of the sparrow's testicular cycle is mediated by the circadian rhythm of photosensitivity [16-19]. Less is known about the circadian and photoperiodic characteristics of the house sparrow inhabiting lower latitudes [19,20]. Therefore, using house sparrows inhabiting a subtropical region (around 27° N), we studied the effects of exposure to natural daylight (NDL) confined to twilight periods on the daily activity rhythm and timing of the termination of testicular activity, the two functions regulated by the circadian system in various bird species including the house sparrow [14-23]. We also compared the effects on the daily activity rhythm at two different times of the year, around the equinoxes (March and September), when daylength is very similar, to determine the influence of seasons, if any, on the effects of twilights on the circadian system that regulates the daily activity rhythm in sparrows.

## Methods

Two experiments were performed on adult male house sparrows (*Passer domesticus*) caught around Lucknow city (27° N, 81° E) and quarantined in an outdoor aviary for 4–6 days.

### Experiment 1: Termination of testicular activity

This experiment compared the timing of testicular regression and concomitant depigmentation of the testosterone-dependent beak color [24] between two groups of sparrows, one of which received NDL during twilights only. The experiment started in the third week of April 2002 and lasted 8.5 weeks. Two groups (n = 6 each) of acclimatized sparrows housed in separate cages (size = 45 × 25 × 25 cm) were kept inside a large outdoor aviary (3.0 × 2.5 × 2.5 m) that received natural illumination from east, north and south. At this time, sparrows had large testes (testis size = 37.60 ± 6.41 mm^3^; n = 12). Group 1 continuously received NDL all day (NDL group), while group 2 was daily removed from the aviary at the time of sunrise, kept in a room under constant darkness (DD) until the time of sunset, and then returned to the aviary. Thus, group 2 birds received NDL only during twilight periods (twilight group). Observations on changes in body mass, beak color and testis size were recorded at the beginning, in the middle, and at the end of the experiment. Body mass of an individual bird was recorded on a top pan balance with an accuracy of 0.1 g. Depigmentation of the beak was assessed as the lightening of its color using a subjective criterion of scores from 0–5, as outlined in Kumar *et al. *[25]. Briefly, the scores were assigned as follows: 0 – bill straw in color (S), 1- bill straw in color but with a little tinge of blackness (ratio- SSS:B), 2- bill slightly blackish in color (ratio- SS:B), 3- bill straw and black in approximately 50:50 patches (ratio- S:B), 4- bill black with very little straw patch left (ratio- S:BB), 5- bill completely black (B). Testicular response was assessed by laparotomy as described by Kumar *et al *[26]. In brief, a small incision was made between the last two ribs on the left flank, the left testis was located within the abdominal cavity with the help of a spatula, and its long (length) and short (width) axes were measured using a caliper with reference to markings on a graph sheet. Testis volume (TV) was calculated using the formula 4/3 π *a b*^*2*^, where *a *and *b *denote half of the length and width, respectively.

### Experiment 2: Effect on the daily activity rhythm

This experiment examined the effects of morning and evening twilight periods on the daily activity pattern of house sparrows exposed to natural illumination. The experiment was conducted twice, around two times of the year when the durations of daylight are the same: the third week of March and the third week of September 2004. Each time, the experiment lasted for six weeks and used a separate group of birds. Eight acclimatized sparrows were housed individually in specially designed activity cages (size [length × width × height] = 50 × 45 × 40 cm) stacked in two columns of 4 each separately within a wooden cabinet (size = 105 × 65 × 165 cm) that was fixed on the east-facing wall outdoor on the first floor underneath a concrete platform (size [length × width] = 168 × 60 cm) such that birds were protected from direct sunlight falling on them from top and sides, and received uninhibited natural illumination from the east. Unlike in experiment 1, the birds were not moved daily to a separate room; they remained undisturbed throughout the experiment (and restriction of daylight to twilights was accomplished by closure of the wooden cabinet). Each activity cage was furnished with two perches and mounted with a passive Infrared Motion Sensor 12 m (40') range (Intellisense XJ 413T, C & K Systems, Conrad Electronic, Germany), which continuously detected, counted and recorded the movement of the bird within its cage. Each sensor was connected to a separate channel, and the recording was done using a software program (Stanford Software Systems, Stanford, CA) run on an IBM-compatible computer. The general activity of a sparrow within its cage was considered to reflect the response of its circadian system [27].

After 2 weeks of initial exposure to NDL, the experiment was completed in two stages. In the first stage, 4 birds were deprived of daylight from sunrise to sunset by shielding the access of sunlight falling on wooden boxes; hence they received NDL during twilight periods only. Simultaneously, the other 4 birds continued receiving NDL conditions all day and served as controls. Two weeks later, in the second stage, lighting conditions of the two halves were swapped: the second half now received NDL during twilight periods only, while the first half received NDL conditions all day and served as controls. Birds were designated as belonging to the NDL group when they received NDL all day, and to the twilight group when they received NDL only during twilights.

The activity of each bird was recorded continuously and plotted as a double-plot actogram, each day being duplicated along the horizontal axis and subsequent days being shown underneath in increasing order, using the program supplied by the Stanford Software Systems. The onset and end of activity during the daytime was calculated with reference to the times of sunrise and sunset, respectively. The phase relationships between activity and the LD cycle were described as the phase angle difference (ψ). The number of movements of each individual was counted and compared. We calculated the period of the rhythm using the chi-square periodogram procedure. Collection and analysis of activity data were performed using the Stanford Software Systems (Stanford, USA). For statistical comparisons, the data from the two stages of the experiment were pooled together. Incomplete data from one bird were excluded from the analysis.

Food and water were available *ad libitum*, and general housing conditions, including handling of experimental animals, were the same as described in several previous publications from our laboratory [19,25-27]. Data are presented as mean ± SE. They were statistically analyzed using 1-way analysis of variance (ANOVA) with or without repeated measures, as appropriate, followed by Newman-Keuls post hoc test, if ANOVA indicated a significance of difference. We employed 2-way ANOVA to compare daily activity counts between NDL and twilight groups; for this, an 11-day segment of actogram of each bird in the two groups was considered, which when analysed resulted in degrees of freedom = 132. Two groups at one time point were compared using Student's t-test. Significance was taken at p < 0.05.

## Results

### Experiment 1: Termination of testicular activity

**Figure 1:**
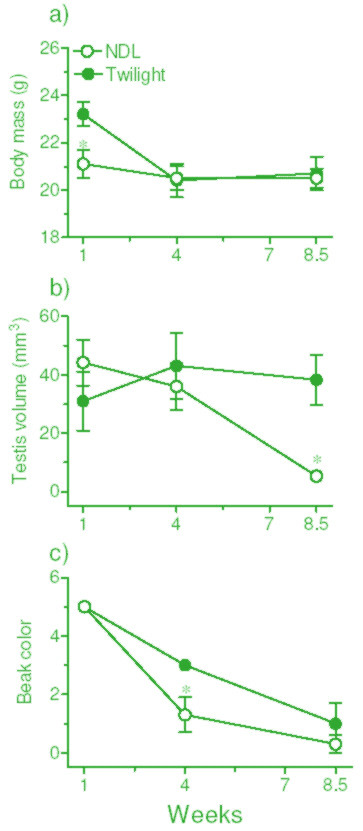
Effects of natural light (NDL) during twilight periods alone on body mass (a), timing of testicular regression (b), and changes in beak color (c). Of two groups of male sparrows (n = 6 each) with large testes housed in the outdoor aviary, beginning in the third week of April 2002 for 8.5 weeks, one group of birds was removed daily from the aviary at the time of sunrise and kept in a room under constant darkness (DD) until sunset, when it was returned to the aviary. The other group remained in the aviary, received NDL all day and served as control. Note testicular regression only in birds that experienced NDL all day.

The results are shown in Figure 1. Testes were of comparable size in the two groups at the beginning of the experiment (Figure 1b), but there was a significant difference (p < 0.05, Student's t-test) in body mass between the two groups (Figure 1a). There was no change in body mass of group 1 birds that received natural light all day (Figure 1a), whereas birds exposed to NDL only during twilights significantly lost body mass within 4 weeks (F _2,10 _= 37.67, p < 0.0001; 1-way RM ANOVA). Interestingly, however, testes underwent significant regression (F _2,10 _= 14.93, p = 0.0010: 1-way RM ANOVA) in birds of group 1 and not of group 2 (Figure 1b). Group 2 birds exposed only to twilight periods maintained large testes until the end of the experiment (Figure 1b). Similarly, testosterone-dependent beak color lightened more rapidly in the NDL than in the twilight group (p < 0.05, week 4; Figure 1c).

### Experiment 2: Effect on daily activity rhythm

**Figure 2:**
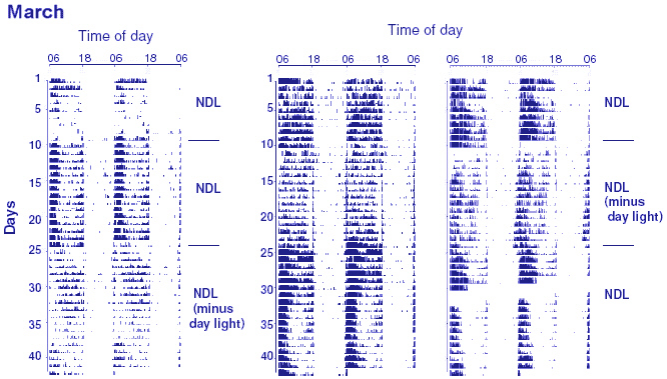
Double plotted activity recordings of three male house sparrows receiving NDL conditions in March. After initial synchronization when they were exposed to NDL all day for 2 weeks, they were exposed to NDL during twilight periods alone in two stages. First, half of them (n = 4) continued receiving NDL all day and served as control (left panel), while the other half (n = 4) was deprived of NDL from sunrise to sunset (NDL minus daylight; middle and right panels). Then, after two weeks, the lighting conditions of the two halves were swapped: the first half received NDL minus daylight (left panel), while the second half received NDL all day and served as control (middle and right panels). The movements of the birds within their cages (size = 60 × 45 × 35 cm) were detected by passive infrared motion sensors, continuously counted and recorded, and analyzed by the software program of the Stanford Software Systems (Stanford, USA). Note freerun between days 15 and 25 in one of the three representative sparrows exposed to twilight periods alone in March (right panel).

**Figure 3:**
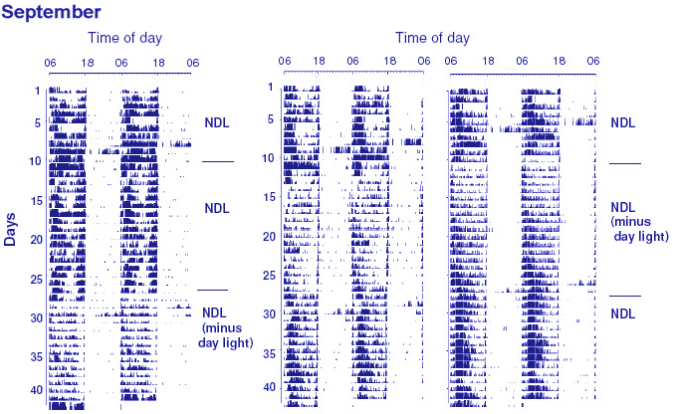
Double plotted activity recordings of three male house sparrows receiving NDL conditions in September. The other details of the experiments were the same as described in Figure 2.

**Figure 4:**
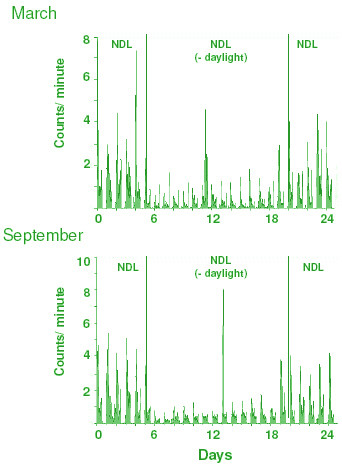
Daily activity profile (counts per minute, mean ± SE, n = 7) of sparrows for the period when they were exposed to NDL during twilight periods alone along with 4 days before and after the twilight exposure (i.e., when they received NDL all day). Note the difference in amplitude of the daily activity rhythm between NDL and NDL minus daylight conditions.

Figures 2 and 3 show the daily activity patterns of three sparrows (Figure 2: March; Figure 3: September), and Figure 4 shows the mean activity profile of 7 sparrows in March and September. At both times of the year, activity rhythms were synchronized to the time of sunrise, with a positive phase angle difference (+ψ) of 0.41 ± 0.05 h (March, Figure 2) or 0.31 ± 0.06 h (September, Figure 3). When deprived of daylight hours from sunrise and sunset, sparrows remained synchronized with a similar phase angle difference: +ψ = 0.80 ± 0.24 h (March) or 0.37 ± 0.06 h (September). ψ in March birds became more variable; in fact 2 of 7 birds lost synchrony and behaved as if they were freerunning with τ = 23.9 h (Figure 2, right panel). The amount of daily activity was significantly greater in NDL than in the twilight condition at both times of the year (March: p = 0.0194, Student's t-test; September: p = 0.0045, Student's t-test). Further, daily activity counts were significantly lower in March than in September both in NDL [F_1,132 _= 24.81, p < 0.0001; 2 way ANOVA] and twilight [F_1,132 _= 10.50, p < 0.0001; 2 way ANOVA] conditions, which indicated a seasonal difference in responsiveness of the circadian system.

## Discussion

There was a decrease in body mass of birds in the twilight group but not in the NDL group (Figure 1a), perhaps because of the low food intake due to insufficient daylight hours available to twilight birds for feeding (sparrows do not eat during absolute darkness, our unpublished observation). Of interest is the result, however, that testes regressed in sparrows exposed to NDL all day, but not in those exposed to NDL only during twilights. Testicular regression was significantly delayed in the latter (twilight) group (Figure 1b). The data on beak color support the observation on testes; beak depigmentation was more rapid in NDL than in the twilight group (Figure 1b and 1c). The lack of exposure to light between twilights, which presumably caused the reduction in body mass (Figure 1a) and activity (Figures 2, 3, and 4), would be expected to advance, not to delay, testicular regression. Availability of food is known to have a direct effect on photoperiod-induced testicular recrudescence [26]. It is also known that, on exposure to long photoperiods at low light intensity, birds regress testes as if they are exposed to short photoperiods [28,29]. Bentley *et al. *[30] found that, in the European starling *(Sturnus vulgaris)*, long day length at low light intensity is perceived as short day length. In the present study, a significant delay in testicular regression among birds in the twilight group may be explained by at least two processes. (1) Light during the twilights falling before and after ~13 h of complete darkness (the interval between sunrise and sunset when the birds were moved to a room in DD) is interpreted differently (twilight group) than when it is continuous with the daylight period (NDL group). A previous study by Kumar *et al. *[31] on migratory blackheaded bunting *(Emberiza melanocephala) *suggested that the subjective interpretation of day and night depends both upon photophase contrast and light intensity. (2) The mediation of photoperiodism by the circadian system in house sparrows [16-18] may involve parametric (light all day) rather than nonparametric (light only in the morning and evening) mechanisms, as reported for the circadian activity rhythm of Syrian hamsters [32].

Consistenly with observations in temperate populations [4,14], the subtropical house sparrows used in the present study showed a bimodal pattern of daytime activity (Figures 2, 3, and 4), which is consistent with the proposition that the morning (M) and the evening (E) activity components reflect M and E oscillatory components of the endogenous circadian system [33]. Deprivation of light from sunrise to sunset had no dramatic effect on the daily activity pattern in either of the seasons, except a relatively larger intra-group variation among March birds. On exposure to twilight periods alone, 2 of 7 March birds freeran albeit with τ very close to 24 h (Figure 2, right panel). On the other hand, all birds in September continued synchronization (Figure 3). Daily activity counts were also significantly lower in March than in September both in the NDL and twilight condition (Figure 4). Such differences in activity pattern between September and March birds are comparable to seasonality in light responsiveness of the endogenous circadian system reported for its temperate population [4,14] and other species [3,34-36]. Could this then mean that the strength of twilight periods of the day as zeitgeber varies with seasons? A recent study on Syrian hamster *(Mesocricetus auratus) *supports this: inclusion of twilights had season- and latitude-dependent effects on several circadian parameters including phase angle difference and period of activity [37]. Our results on the sparrow activity rhythm are not directly comparable with those of the hamster studies [32,37] because the hamster studies used an artificial lighting paradigm consisting of LD cycle plus simulated twilight periods with a rectangular LD cycle as control. We instead used a natural lighting paradigm, which directly tested the effects of twilight timings alone, and not in conjunction with daily light period.

In summary, this is the first study that examined the role of natural light restricted to twilights in the regulation of photoperiod-induced testicular response and the daily activity rhythm in a vertebrate species. Exposure to natural daylight restricted to twilights delayed the timing of testicular regression and concomitant depigmentation of the beak, but did not affect the daily activity rhythm in male house sparrows. Thus, we showed a differential effect of twilight times in the regulation of two circadian functions, namely photoperiodism and activity rhythm, in subtropical house sparrows.

## Conclusion

Natural light restricted to twilights can serve as a cue for the regulation of circadian activity rhythms but not for the circadian-mediated photoperiodic regulation of the testicular cycle in the house sparrow. This finding raises the possibility that separate circadian processes govern these two circadian functions in the house sparrow.

## Competing interests

The author(s) declare that they have no competing interest.

## Authors' contributions

AKT and SR carried out the experiments and prepared the initial draft of the manuscript. VK supervised the experiments and produced the final version of the manuscript. The study was conceived by VK but planned by all three authors. All the authors approved the final version of the manuscript.

## References

[B1] Rani S, Singh S, Kumar V, Kumar V (2002). Light sensitivity of the biological clock. Biological Rhythms.

[B2] Roenneberg T, Foster RG (1997). Twilight times: Light and the circadian system. Photochemistry Photobiology.

[B3] Daan S, Aschoff J (1975). Circadian rhythms of locomotor activity in captive birds and mammals: Their variation with season and latitude. Oecologica.

[B4] Binkley S, Mosher K (1992). Activity rhythms in house sparrows exposed to natural lighting for one year. J Interdiscipl Cycle Res.

[B5] Aschoff J, Weaver R (1965). Circadian rhythms of finches in light-dark cycles with interposed twilights. Comp Biochem Physiol.

[B6] Reitveld WJ, Tordoir WEM (1965). The influence of twilight duration on locomotor activity of rabbits. Acta Physiol Pharmacol Neerl.

[B7] Kavanau JL (1962). Twilight transitions and biological rhythmicity. Nature.

[B8] Kavanau JL (1962). Activity pattern on regimes employing artificial twilight transitions. Experientia.

[B9] Kavanau JL (1968). Activity and orientational responses of white footed mice to light. Nature.

[B10] Kavanau JL (1969). Influences of light on activity of small animals. Ecology.

[B11] Kavanau JL, Ramos J (1970). Roadrunners: Activity of captive individuals. Science.

[B12] Hammond RD, Naylor E (1977). Effects of dusk and dawn on locomotor activity rhythms in the Norway lobster, *Nephrops norvegicus*. Marine Biol.

[B13] Boulos Z, Macchi M, Houptet TA, Terman M (1996). Photic entrainment in hamsters: Effects of simulated twilights and nest box availability. J Biol Rhythms.

[B14] Binkley S (1990). The clockwork sparrow: Time, clock and calendars in biological organisms.

[B15] Brandstätter R, Kumar V, Van't Hof TJ, Gwinner E (2000). Seasonal variations of *in vivo *and *in vitro *melatonin production in a passeriform bird, the house sparrow (*Passer domesticus*). J Pineal Res.

[B16] Menaker M, Eskin A (1967). Circadian clock in photoperiodic time measurement: A test of the Bünning hypothesis. Science.

[B17] Murton RK, Lofts B, Westwood NJ (1970). Manipulation of photorefractoriness in the house sparrow *(Passer domesticus) *by circadian light regimes. Gen Comp Endocr.

[B18] Farner DS, Donham RS, Lewis RA, Mattock PW, Darden TR, Smith JP (1977). The circadian component in the photoperiodic mechanism of the house sparrow, *Passer domesticus.*. Physiol Zool.

[B19] Trivedi AK, Rani S, Kumar V (2005). Differential responses of the photoperiodic clock in two passerine birds possessing strongly self-sustained circadian system. Chronobiol Int.

[B20] Trivedi AK (2004). Seasonal responses of house sparrow (*Passer domesticus*) Linneaus at 27°N. Ph D Thesis.

[B21] Kumar V, Follett BK (1993). The nature of photoperiodic clock in vertebrates. Proc Zool Soc Calcutta J B S Haldane Comm Vol.

[B22] Dawson A, King VM, Bentely GE, Ball GF (2001). Photoperiodic control of seasonality in birds. J Biol Rhythms.

[B23] Kumar V, Singh BP, Rani S (2004). The bird clock: A complex, multi-oscillatory and highly diversified system. Biol Rhythms Res.

[B24] Lofts B, Murton RK, Thearle RJP (1973). The effects of testosterone propionate and gonadotropins on the bill pigmentation and testes of the house sparrow *(Passer domesticus)*. Gen Comp Endocrinol.

[B25] Kumar V, Singh S, Misra M, Malik S, Rani S (2002). Role of melatonin in photoperiodic time measurement in the migratory redheaded bunting *(Emberiza bruniceps) *and the non-migratory Indian weaver bird *(Ploceus philippinus).*. J Exp Zool.

[B26] Kumar V, Singh S, Misra M, Malik S (2001). Effects of duration and time of food availability on photoperiodic responses in the migratory male blackheaded bunting *(Emberiza melanocephala)*. J Exp Biol.

[B27] Malik S, Rani S, Kumar V (2004). Wavelength dependency of light-induced effects on photoperiodic clock in the migratory blackheaded bunting *(Emberiza melanocephala)*. Chronobiol Int.

[B28] Kumar V (1986). The photoperiodic entrainment and induction of reproductive rhythms in male blackheaded bunting (*Emberiza melanocephala*). Chronobiol Int.

[B29] Kumar V (1988). Investigations of photoperiodically induced fattening in migratory blackheaded bunting (*Emberiza melanocephala*) (Aves). J Zool.

[B30] Bentley GE, Goldsmith AR, Dawson A, Briggs C, Pemberton M (1998). Decreased light intensity alters the perception of day length by male European starlings *(Sturnus vulgaris).*. J Biol Rhythms.

[B31] Kumar V, Kumar BS, Singh BP (1992). Photostimulation of blackheaded bunting: Subjective interpretation of day and night depends both upon photophase contrast and light intensity. Physiol Behav.

[B32] Boulos Z, Macchi M, Terman M (2002). Twilights widen the range of photic entrainment in hamsters. J Biol Rhythms.

[B33] Daan S, Albrecht U, Horst van der GTJ, Illnerova H, Roenneberg T, Wehr TA, Schwartz WJ (2001). Assembling a clock for all seasons: Are there M and E oscillators in the genes?. J Biol Rhythms.

[B34] Pohl H (1972). Seasonal change in light sensitivity in *Carduelis flammea*. Naturwissenschaften.

[B35] Gwinner E (1975). Effect of season and external testosterone on the free-running circadian activity rhythm of European starlings *(Sturnus vulgaris)*. J Comp Physiol.

[B36] Jain N (1993). Strategies for endogenous programming in the migratory blackheaded bunting, *Emberiza melanocephala *Scopoli. PhD Thesis.

[B37] Boulos Z, Macchi M (2005). Season and Latitude dependent effects of simulated twilights on circadian entrainment. J Biol Rhythms.

[B38] Rani S, Singh S, Kumar V (2005). The pineal clock affects behavioral circadian rhythms but not photoperiodic induction in the Indian weaver bird (*Ploceus philippinus*). J Ornithol.

